# Prenatal Diagnosis, Ultrasound Findings, and Follow‐Up Evaluation of 16p13.11 Deletion and Duplication Syndromes: Preliminary Assessment of Fetal Genotype–Phenotype

**DOI:** 10.1002/jcla.70051

**Published:** 2025-06-19

**Authors:** Xiaojin Luo, Liping Wu, Jinshuang Song, Jinmao Xu, Ruchun Huang, Hongyan Niu, Fei Zhou, Yuanyuan Pei, Weiqiang Liu, Fengxiang Wei

**Affiliations:** ^1^ The Genetics Laboratory Longgang District Maternity & Child Healthcare Hospital of Shenzhen City (Longgang Maternity and Child Institute of Shantou University Medical College) Shenzhen Guangdong China

**Keywords:** 16p13.11 deletion, 16p13.11 duplication, prenatal diagnosis, SNP array, ultrasonography

## Abstract

**Objective:**

To analyze the ultrasound findings, single nucleotide polymorphism microarray (SNP array) results, pregnancy outcomes, and follow‐up information of fetuses with 16p13.11 deletion or duplication.

**Methods:**

This retrospective study collected data from 14 fetuses diagnosed with 16p13.11 deletion and 12 fetuses with 16p13.11 duplication. The study involved a review and analysis of maternal demographics, ultrasound findings, SNP‐array results, pregnancy outcomes, and follow‐up information.

**Results:**

The copy number variations (CNVs) observed ranged in size from 0.92 to 2.85 Mb for 16p13.11 deletions and from 0.89 to 2.84 Mb for duplications. These CNVs included seven OMIM genes: *NDE1*, *MYH11*, *ABCC1*, *XYLT1*, *MARF1*, *CEP20*, and *ABCC6*. Among the 14 fetuses with 16p13.11 deletions, seven (50.0%, 7/14) revealed abnormalities in ultrasound findings. Cardiovascular anomalies were present in five cases (35.7%, 5/14); two cases (14.3%, 2/14) showed lateral ventricular widening. Cases 2 and 14 were particularly noteworthy, as both presented complex malformations affecting multiple organs. Among the 12 fetuses with duplications, five cases (41.7%, 5/12) exhibited ultrasound abnormalities. Of these, three cases (25.0%, 3/12) presented with cardiovascular abnormalities; two cases (16.7%, 2/12) displayed widened lateral ventricles. Case 25 was particularly distinct, featuring complex multiorgan malformations that included widened lateral ventricles, tricuspid regurgitation, and a right ear malformation. Of the eight fetuses with 16p13.11 deletions whose pregnancies were continued, three exhibited neurodevelopmental abnormalities. Ten fetuses with 16p13.11 duplications that were followed up, two cases showed neurodevelopmental abnormalities.

**Conclusion:**

Our study expanded the clinical phenotype spectrum of fetuses with 16p13.11 deletion and duplication and conducted a preliminary evaluation of prenatal ultrasound findings in conjunction with postnatal clinical phenotypes. The primary manifestations observed in fetuses with 16p13.11 deletion and duplication are likely to be cardiovascular malformations and widened lateral ventricles.

## Graphical abstract

What do the results of this study add?

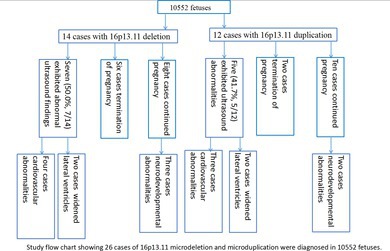

We expanded the clinical phenotype spectrum of fetuses with 16p13.11 deletion and duplication and conducted a preliminary evaluation of prenatal ultrasound findings in conjunction with postnatal clinical phenotypes. The primary manifestations observed in fetuses with 16p13.11 deletion and duplication are likely to be cardiovascular malformations and widened lateral ventricles. The deletion and duplication of 16p13.11 are closely linked to neural development, necessitating careful monitoring of the fetus after birth.

## 1 Introduction

Deletion and duplication of chromosomal segments, which alter the dosage of genetic material, are the primary causes of chromosomal deletion and duplication syndromes. These syndromes are strongly linked to intellectual disabilities, multiple malformations, and neurodevelopmental abnormalities [1]. Approximately 5% of the human genome is composed of low‐copy repeats (LCRs) within chromosomes. Nonallelic homologous recombination (NAHR) between these LCRs can result in abnormal genomic rearrangements. Many deletion and duplication syndromes, including the 16p13.11 deletion syndrome, have been identified as being caused by these LCRs [2]. Children with 16p13.11 deletion syndrome commonly display clinical phenotypes such as language delays, intellectual disabilities, epilepsy, autism, facial malformations, and urogenital system abnormalities. Similarly, 16p13.11 duplication syndrome is closely associated with intellectual disabilities, autism spectrum disorders, and certain impulsive and aggressive behaviors [3], but lacked specificity [4]. In recent years, the deletion or duplication syndrome in the 16p13.11 region has garnered significant attention from clinical and laboratory professionals due to its strong association with neurocognitive aspects. Previous studies have thoroughly elucidated the correlation between copy number variation and phenotype in children or adolescents with 16p13.11 deletion or duplication [5]. However, in prenatal diagnosis, cases involving 16p13.11 deletion or duplication are typically reported either as individual cases or within relatively small cohort populations [6]. Consequently, there is a notable lack of systematic discussion on clinical features, pregnancy outcomes, and prognosis. Additionally, genetic counseling faces significant challenges due to the diversity of phenotypes and incomplete penetrance [7]. This study, based on a relatively large cohort of fetuses with 16p13.11 deletions and duplications, explores clinical manifestations, pregnancy outcomes, and follow‐up information. It aims to provide a broader understanding of genotype–phenotype correlations for clinical consultants involved in prenatal diagnosis.

## 2 Subjects and Methods

### 2.1 Subjects

Cases were retrospectively collected from October 2018 to June 2024 at Shenzhen Longgang District Maternal and Child Health Hospital due to indications including advanced maternal age, ultrasound abnormalities (such as fetal structural abnormalities or soft marker abnormalities), high risk of serological screening results, and abnormal results of noninvasive prenatal testing. The study was approved by the Ethics Committee of Longgang Maternal and Child Healthcare Hospital (LGFYYXLL‐028). All participants were fully informed about the research purpose and experimental process, and signed informed consent. The study focused exclusively on cases of pure 16p13.11 deletion and duplication, excluding any cases with other chromosomal abnormalities. SNP array testing was conducted on the parents of the fetuses with 16p13.11 deletion and duplication to determine whether the genetic variations were inherited or de novo. Prenatal ultrasound diagnosis followed the International Society of Ultrasound in Obstetrics and Gynecology (ISUOG) guidelines for second and third trimester fetal ultrasound scan [8]. The examination was conducted by two experienced ultrasound technicians, both certified in fetal scanning, using the Voluson E8 and Voluson E10 color Doppler ultrasound systems (GE Healthcare, USA) equipped with convex array probes, operating at a frequency of 3–5 MHz.

## 3 Methods

### 3.1 SNP Array

Sterile samples of 10 mL of amniotic fluid or 2 mL of cord blood were collected, centrifuged, and subsequently precipitated. DNA extraction was performed using the QIAamp DNA Kit (QIAGEN, Germany). The short tandem repeat (STR) method was employed to detect and confirm the absence of maternal blood contamination. The genomic DNA was enzymatically digested into short fragments, which were then ligated and amplified via PCR. The PCR products were purified using magnetic beads. These purified products were further fragmented into 25–125 bp pieces and labeled with biotin. The labeled fragments were mixed with a hybridization solution, denatured, and hybridized onto a chip. Following hybridization, the chip was washed and stained. The Affymetrix CytoScan 750 K GeneChip platform (Thermo Scientific, USA) was utilized for scanning the chip.

### 3.2 Data Analysis

All detection data were processed and analyzed using the Chromosome Analysis Suite (ChAS) V4.0 software. This software is specifically designed to identify clinically relevant genes and copy number variations (CNVs) with a genomic resolution exceeding 100 kb. The classification of CNVs was conducted in accordance with the guidelines provided by the American College of Medical Genetics (ACMG). Additionally, resources such as the UCSC Genome Browser, Online Mendelian Inheritance in Man (OMIM), Clinical Genome Resource (ClinGen), Database of Chromosomal Imbalance and Phenotype in Humans Using Ensemble Resources (DECIPHER), Database of Genomic Variants (DGV), and internal databases were utilized. According to ACMG guidelines, CNV classifications are categorized into pathogenic/likely pathogenic, variants of uncertain significance (VOUS), and likely benign/benign.

### 3.3 Follow‐Up

All 26 cases underwent follow‐up, during which we monitored their pregnancy outcomes and recorded the delivery conditions of the pregnant women, as well as the growth and development of the newborns. Follow‐ups occurred at least biannually. At the latest follow‐up, the ages of the children varied from 2 months to 5 years and 1 month.

## 4 Results

### 4.1 Analysis of SNP Array Results

During the study period, a total of 10,552 fetuses underwent prenatal SNP array testing. Of these, 14 cases (0.15%, 16/10,552) were identified with 16p13.11 deletion and 12 cases (0.11%, 12/10,552) with 16p13.11 duplication. The median maternal age was 29 ± 6 years, ranging from 22 to 38 years, and the median gestational age at the time of prenatal diagnosis was 21 ± 5 weeks, ranging from 16 to 26 weeks. The fetuses with deletions, Cases 1–14, exhibited deletion fragments ranging in size from 0.92 to 2.85 Mb. The fetuses with duplications, Cases 15–26, had duplication fragments ranging from 0.89 to 2.84 Mb. The overlapping region of 16p13.11 deletion and duplication across the 26 cases is primarily located between 15.5 and 16.3 Mb, encompassing 14 protein‐coding genes, including *NDE1*, *MYH11*, *ABCC1*, *XYLT1*, *MARF1*, *CEP20*, and *ABCC6*, with *MYH11* classified as a haploinsufficiency gene. Parental origin verification was performed for 23 fetuses, including 13 cases with 16p13.11 deletions and 10 cases with 16p13.11 duplications. Of these, nine cases were identified as de novo, including seven deletions and two duplications. Fourteen cases were inherited from a parent, comprising six deletions and eight duplications (Table 1).

**TABLE 1:** Prenatal indications, SNP array results, and follow‐up information of 26 cases with 16p13.11 deletion and duplication.

Case	Age	GW	High risk/ultrasound findings	SNP array results	Size (Mb)	Inheritance	Outcome	Follow‐up information
1	30	23	TOF, SUA	arr[hg19] 16p13.11 (14929070‐16409046)x1	1.48	Denovo	TOP	N/A
2	30	24	Right kidney absent, VSD, strephenopodia	arr[hg19] 16p13.11 (15324776‐18172468)x1	2.85	Denovo	TOP	N/A
3	37	19	Left LVW (10.7 mm), AMA	arr[hg19] 16p13.11 (15053116‐16303324)X1	1.28	Denovo	Live birth	CS, no visible abnormalities
4	29	17	NIPT indicated chromosome 16 deletion	arr[hg19] 16p13.11 (15044228‐16602286)X1	1.57	Inherited $\left(\text{maternal}\right)$	Live birth	CS, language development delay
5	36	16	AMA, HROSS	arr[hg19] 16p13.11 (14968855‐16194578)X1	1.23	Unknown	Live birth	ND, no visible abnormalities
6	28	18	NIPT indicated chromosome 16 deletion	arr[hg19] 16p13.11 (15492317‐18312776)X1	2.82	Denovo	TOP	N/A
7	24	18	HROSS	arr[hg19] 16p13.11 (14969632‐16525348)X1	1.56	Inherited $\left(\text{maternal}\right)$	Live birth	ND, language development delay, epilepsy
8	38	19	AMA, HROSS	arr[hg19] 16p13.11 (15044624‐16194613)X1	1.15	Inherited $\left(\text{paternal}\right)$	Live birth	ND, no visible abnormalities
9	29	25	VSD	arr[hg19] 16p13.11 (15041186‐16763246)X1	1.72	Denovo	TOP	N/A
10	33	20	NIPT indicated chromosome 16 deletion	arr[hg19] 16p13.11 (15124598‐16732254)X1	1.61	Inherited $\left(\text{maternal}\right)$	Live birth	ND, poor communication skill, hypotonia, motor incoordination
11	36	26	GR, VSD	arr[hg19] 16p13.11 (15481265‐16892236)X1	1.41	Denovo	TOP	N/A
12	25	21	HROSS, LVW (11.0 mm)	arr[hg19] 16p13.11 (15472265‐16398764)X1	0.92	Inherited $\left(\text{maternal}\right)$	Live birth	ND, no visible abnormalities
13	23	18	NIPT indicated chromosome 16 deletion	arr[hg19] 16p13.11 (15122156‐16305642)X1	1.18	Denovo	Live birth	ND, no visible abnormalities
14	32	24	Aortic stenosis, horseshoe kidney, cleft lip	arr[hg19] 16p13.11 (14936625‐16725268)X1	1.79	Inherited $\left(\text{maternal}\right)$	TOP	N/A
15	25	24	NIPT indicated chromosome 16 duplication	arr[hg19] 16p13.11 (15058821‐16572403)x3	1.52	Inherited $\left(\text{paternal}\right)$	Live birth	ND, poor language expression ability and concentration
16	22	22	NIPT indicated chromosome 16 duplication	arr[hg19] 16p13.11 (15499445‐16989059)x3	1.49	Unknown	Live birth	CS, no visible abnormalities
17	32	26	LVW (11.3 mm), GR	arr[hg19] 16p13.11p12.3 (15338152‐18172468)x3	2.84	Denovo	TOP	N/A
18	28	17	HROSS	arr[hg19] 16p13.11 (15139129‐16548596)x3	1.41	Inherited $\left(\text{maternal}\right)$	Live birth	CS, hyperactivity, language and motor development delay
19	28	25	Atrial septal defect	arr[hg19] 16p13.11 (15059764‐16809046)x3	1.75	Unknown	Live birth	ND, surgical treatment
20	29	18	NIPT indicated chromosome 16 duplication	arr[hg19] 16p13.11 (15319278‐17152486)x3	1.84	Inherited $\left(\text{maternal}\right)$	Live birth	ND, newborn with hearing loss in the right ear
21	29	23	VSD	arr[hg19] 16p13.11 (15054310‐16349225)x3	1.29	Inherited $\left(\text{paternal}\right)$	Live birth	ND, surgical treatment
22	22	21	NIPT indicated chromosome 16 duplication	arr[hg19] 16p13.11 (15338153‐17165043)x3	1.83	Inherited $\left(\text{maternal}\right)$	Live birth	ND, no visible abnormalities
23	37	24	AMA, HROSS	arr[hg19] 16p13.11 (15064426‐16278133)x3	1.21	Inherited $\left(\text{paternal}\right)$	Live birth	ND, no visible abnormalities
24	29	22	HROSS, SUA	arr[hg19] 16p13.11 (14892976‐16327887)x3	1.43	Denovo	Live birth	CS, no visible abnormalities
25	33	26	LVW (11.8 mm), tricuspid regurgitation, right ear malformation	arr[hg19] 16p13.11 (14893246‐16737682)X3	1.84	Inherited $\left(\text{maternal}\right)$	TOP	N/A
26	25	23	HROSS	arr[hg19] 16p13.11 (15481748‐16372403)x3	0.89	Inherited $\left(\text{paternal}\right)$	Live birth	CS, no visible abnormalities

Abbreviations: AMA, advanced maternal age; CS, cesarean section; GR, growth retardation; GW, gestational week; HROSS, high risk of serum screening; LVW, lateral ventricle widened; N/A, not applicable; ND, normal delivery; NIPT, noninvasive prenatal testing; SUA, single umbilical artery; TOF, tetralogy of fallot; TOP, termination of pregnancy; VSD, ventricular septal defect.

### 4.2 Ultrasonic Manifestations of Fetuses With 16p13.11 Deletion and Duplication

#### 4.2.1 Fetuses With 16p13.11 Deletion

**FIGURE 1:**
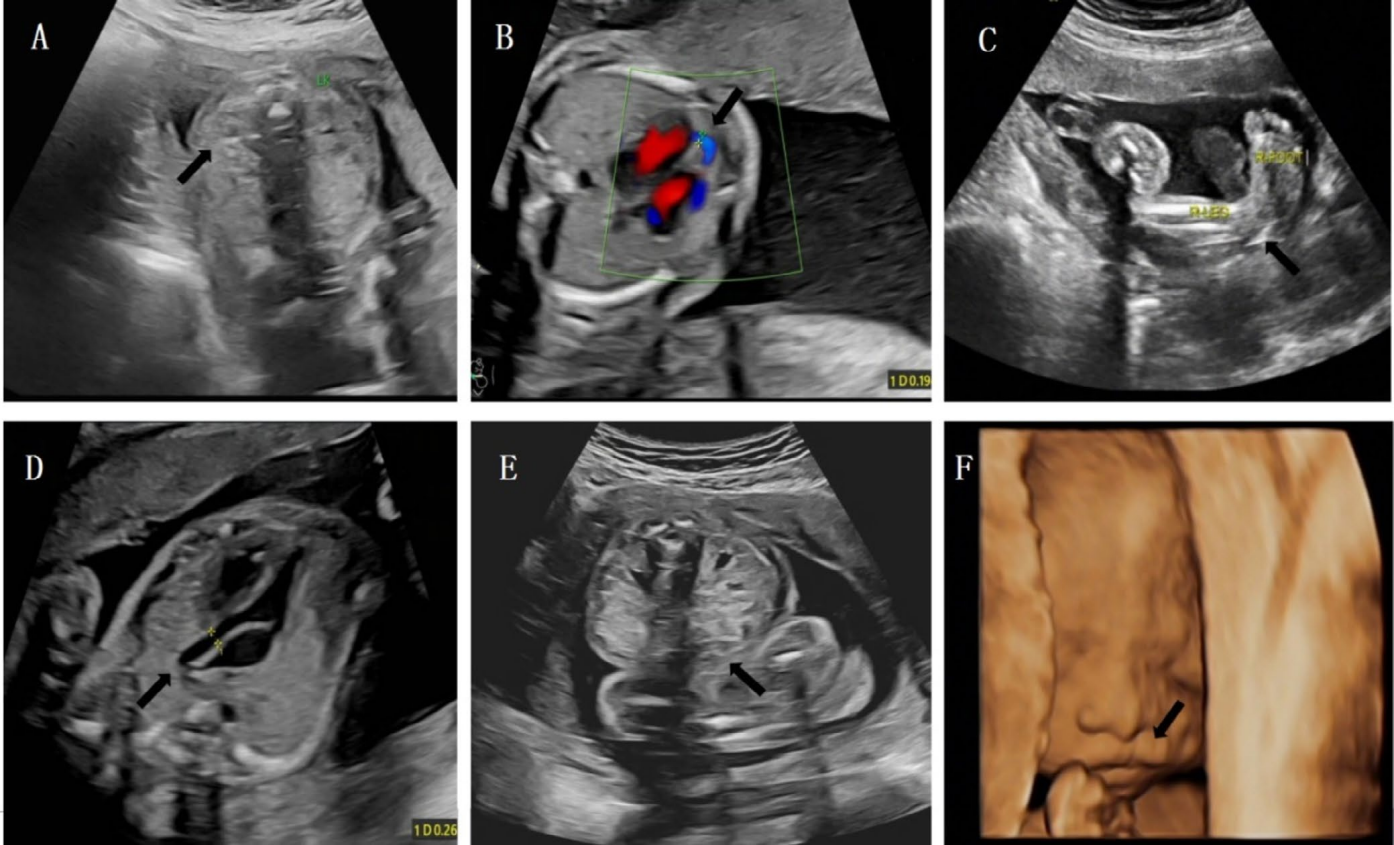
Ultrasound findings of Case 2 (A–C) and Case 14 (D–F) showed multiple structural malformations. *Note:* (A) Absence of the right kidney compared with the left kidney; (B) Ventricular septal defect; (C) Strephenopodia; (D) Aortic stenosis; (E). Horseshoe kidney; (F). Ultrasonic indicates cleft lip at 23 weeks.

Of the 14 fetuses with deletions, seven (50.0%, 7/14) exhibited abnormal ultrasound findings. Among these, five cases (35.7%, 5/14) presented with cardiovascular abnormalities, including three cases of ventricular septal defects, one case of tetralogy of Fallot, and one case of aortic stenosis. Additionally, two cases (14.3%, 2/14) showed widened lateral ventricles. Other findings included one case of strephenopodia, one case with an absent right kidney, one case with horseshoe kidney, one case with a cleft lip, and one case with growth retardation (Tables 1 and 2). Cases 2 and 14 were notable for their complex malformations involving multiple organs. Case 2, which was de novo, predominantly featured strephenopodia, a ventricular septal defect, and an absent right kidney (Figure 1A–C). Case 14, inherited from the mother, mainly involved aortic stenosis, horseshoe kidney, and cleft palate (Figure 1D–F). The mother exhibited a normal clinical phenotype. She described herself as an introvert who was resistant to school and had only completed her junior high school education. Six of the 14 deletion cases were inherited from their parents. Specifically, Case 8 was inherited from the mother, who presented with a normal clinical phenotype and intelligence; however, a renal ultrasound scan revealed multiple cysts in both kidneys. The parents of the other cases exhibited normal clinical phenotypes, and their ultrasound examinations showed no abnormalities.

**TABLE 2:** Ultrasound features of fetuses with 16p13.11 deletion.

Ultrasound findings	Cases	
Cardiovascular abnormalities	5	41.7%
Ventricular septal defects	3	25.0%
Aortic stenosis	1	8.3%
Tetralogy of fallot	1	8.3%
Widened lateral ventricles	2	16.7%
Strephenopodia	1	8.3%
Absence of right kidney	1	8.3%
Horseshoe kidney	1	8.3%
Cleft lip	1	8.3%
Growth retardation	1	8.3%
Total	12	

### 4.3 Fetuses With 16p13.11 Duplication

**FIGURE 2:**
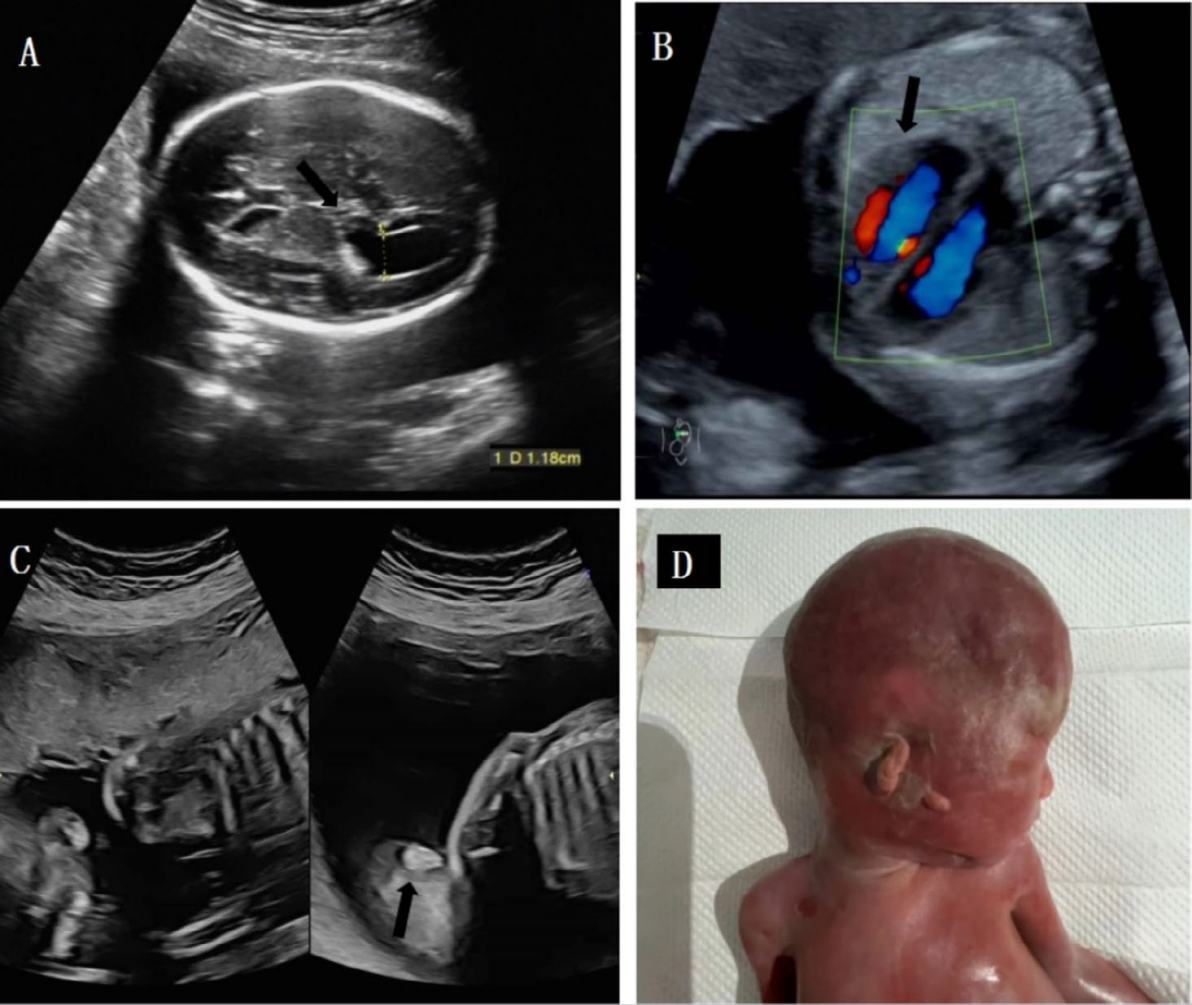
Prenatal and postnatal features of Case 25 showed multiple structural malformations. *Note:* (A) Widened lateral ventricles, the width is 11.8 mm; (B). Tricuspid regurgitation; (C). Right ear malformation; (D). Right ear malformation was identified after termination of pregnancy at 26 weeks.

Of the 12 fetuses with duplications, five (41.7%, 5/12) exhibited ultrasound abnormalities. Among these, four cases (33.3%, 4/12) presented with cardiovascular abnormalities, including atrial septal defects, ventricular septal defects, and tricuspid regurgitation. Additionally, two cases (16.7%, 2/12) showed widened lateral ventricles. There was also one case of growth retardation and one case of right ear malformation (Tables 1 and 3). Case 25 is particularly noteworthy; the mutation is maternally inherited and includes complex multiorgan malformations such as widened lateral ventricles, tricuspid regurgitation, and right ear malformation (Figure 2A–D). His mother has been diagnosed with bipolar disorder and has a history of postpartum depression. Despite her resistance to formal education, she communicates well with others. Eight duplication cases were inherited from parents who exhibited normal clinical phenotypes, and no abnormalities were detected on ultrasound examinations.

**TABLE 3:** Ultrasound features of fetuses with 16p13.11 duplication.

Ultrasound findings	Cases	
Cardiovascular abnormalities	3	42.9%
Atrial septal defects	1	14.3%
Ventricular septal defects	1	14.3%
Tricuspid regurgitation	1	14.3%
Widened lateral ventricles	2	28.6%
Growth retardation	1	14.3%
Right ear malformation	1	14.3%
Total	7	

### 4.4 Pregnancy Outcome and Follow‐Up Information

Of the 14 fetuses with 16p13.11 deletions, six families opted for termination following genetic counseling, while eight chose to continue the pregnancy. Postnatal follow‐up revealed that three of the cases exhibited neurodevelopmental abnormalities. Case 4, who is 4 years and 7 months old, exhibits language development delay. Case 7, aged 3 years and 8 months, has experienced both a language development delay and seizure. Case 10, who is 2 years and 1 month old, presents with poor motor coordination, hypotonia, and poor communication skills. Of the 12 fetuses diagnosed with 16p13.11 duplications, two families opted for termination following genetic counseling, while 10 chose to continue the pregnancy. Follow‐up on these 10 cases revealed neurodevelopmental abnormalities in two cases. Case 15, who is currently 4 years and 5 months old, demonstrates weak language skills and poor concentration. Case 18, aged 3 years and 6 months, exhibits language and motor development delay. Case 19 and Case 21 underwent cardiac surgery after birth and currently have a favorable prognosis. For Case 20, the newborn was screened after birth and found to have a right ear injury, but no other neurodevelopmental abnormalities were found.

## 5 Discussion

The clinical phenotype spectrum associated with 16p13.11 copy number variations is complex and diverse, primarily involving neurodevelopmental abnormalities. Therefore, identifying the genetic cause is crucial for assessing prognosis and guiding decisions during pregnancy [7, 9]. The 16p13.11 region is divided into three single‐copy sequence intervals, each bordered by low‐copy repeats (LCRs). Interval II (chr16:15.48 ~ 16.32 Mb) is a critical region for genomic variation. The typical 16p13.11 deletion or duplication encompasses 14 protein‐coding genes, including *NDE1*, *MYH11*, *ABCC1*, *XYLT1*, *MARF1*, *CEP20*, and *ABCC6*. *MYH11* (OMIM: 160745) encodes the primary contractile protein in smooth muscle cells and is associated with familial thoracic aortic aneurysms and dissections. Its heterozygous mutation leads to a significant reduction in the elasticity of the aortic wall [10]. Additionally, reports [11] indicate that coding variations and CNVs in *MYH11* can contribute to both hereditary and sporadic vascular diseases, including familial and sporadic thoracic aortic aneurysms, as well as other sporadic human vascular diseases. A study cohort focusing on the 16p13.11 duplication phenotype spectrum revealed that among 206 children or adults, 15% were diagnosed with congenital heart anomalies, with septal defects being the most common [12]. In our study, 35.7% (5/14) of fetuses with 16p13.11 deletions exhibited cardiovascular system abnormalities, including three cases of ventricular septal defects, one case of tetralogy of Fallot, and one case of aortic stenosis. Additionally, 33.3% (4/12) of fetuses with 16p13.11 duplications presented with cardiovascular abnormalities. It has been reported that cardiovascular malformations may be one of the prenatal ultrasound phenotypes of 16p13.11 duplication [4]. Our research suggests that prenatal ultrasound findings of cardiovascular abnormalities may be closely associated with 16p13.11 deletions and duplications.

*ABCC1* (OMIM: 158343) and *ABCC6* (OMIM: 603234) are both members of the ATP‐binding cassette transporter superfamily, encoding multidrug resistance proteins 1 and 6, respectively. Evidence suggests that autosomal dominant hereditary deafness is caused by a heterozygous mutation in the *ABCC1* gene on chromosome 16p13.11 [13]. The deletion of chromosome 16p13.11, where this gene is located, can lead to general developmental disorders, including delayed speech development, learning difficulties, and hearing impairment, some of which may be accompanied by inner ear malformations [14]. The function of the *ABCC6* gene remains unclear, and its variations may be associated with cardiovascular damage and neuropsychiatric abnormalities [15]. In our study, one case of 16p13.11 duplication showed right ear malformation on ultrasound, and another case with 16p13.11 duplication, although prenatal ultrasound did not detect phenotypic malformation, exhibited right ear hearing loss during the follow‐up period. However, to clearly elucidate the correlation between deafness, ear malformations, and copy number variations in the 16p13.11 region, further observation of more cases and additional mechanistic research are required.

The *NDE1* gene (OMIM: 609449) is considered a candidate gene associated with neurocognitive phenotypes [16]. It encodes a nuclear distribution protein located in the centrosome and plays a crucial role in microtubule organization, mitosis, and neuronal migration [17]. Previous studies have shown that variations in the dosage of *NDE1* may lead to secondary genomic mutations in cortical neurons, which underlie various developmental neurological disorders [18]. Additionally, a 2017 study indicated that miR‐484 is a dosage‐sensitive candidate gene in this region. miR‐484 (15643294–15,643,372) regulates cortical neurogenesis by inhibiting the translation and mRNA stability of Protocadherin 19 (PCDH19). Furthermore, considering that the influence of miRNAs may be regulated by polymorphisms in their target regions, the disease mechanisms mediated by them can also explain low expression and phenotypic variability [19]. In our study, although prenatal ultrasound did not detect phenotypic abnormalities in three fetuses with 16p13.11 deletions and two fetuses with 16p13.11 duplications, neurocognitive impairments were found during the follow‐up period. This suggests that *NDE1* is closely related to neuropsychiatric abnormalities associated with 16p13.11 deletions and duplications.

The penetrance rates of 16p13.11 deletions or duplications are 13.1% and 8.5%, respectively [3]. The 16p13.11 deletion is believed to be associated with neurocognitive developmental disorders because this region contains a haploinsufficiency gene, which is commonly classified as a pathogenic CNV in clinical practice [20]. Patients with deletions in the 16p13.11 region displayed developmental delays, intellectual disabilities, and autism. There are also case reports featuring multiple malformations, cardiovascular abnormalities, sporadic epilepsy, and microcephaly [12]. Atli [21] conducted a study involving 1298 cases of unexplained intellectual disability and/or multiple congenital anomalies and discovered a clustering of 16p13.11 deletions among the patients. Allach [22] analyzed the 16p13.11 fragment in 1027 patients and discovered that deletions were significantly associated with mental retardation and/or multiple congenital anomalies. However, previous research primarily involves children or adolescents, with fewer studies focusing on prenatal fetuses with 16p13.11 deletions. There are a few case reports showing that fetuses with 16p13.11 deletions exhibit ultrasound findings such as ventriculomegaly, increased nuchal translucency, and cardiovascular abnormalities [23]. In this study, ultrasound abnormalities were observed in seven out of 14 cases with 16p13.11 deletions, with cardiovascular abnormalities and lateral ventriculomegaly being the most common features. Additionally, we identified two cases of complex malformations that had not been previously reported, with clinical manifestations including cardiovascular anomalies, absence of the right kidney, strephenopodia, horseshoe kidney, and cleft palate. Our research further elucidates the complexity and diversity of 16p13.11 duplication in fetuses.

Compared with the deletion of 16p13.11, the duplication of 16p13.11 has a lower penetrance and is associated with developmental delays, learning difficulties, and behavioral abnormalities [12]. It represents a neuro‐susceptibility locus characterized by incomplete penetrance and variable expression. Other clinical features, such as cardiac abnormalities, have also been reported [9]. Research indicates that the occurrence of 16p13.11 duplication does not significantly differ between the normal population and individuals with neurodevelopmental abnormalities [24]. Some studies suggest that it may be related to clinical phenotypes of neurodevelopmental symptoms such as mental retardation, autism, and language delays. A recent report showed that patients with 16p13.11 duplication have a significantly increased risk of cardiovascular disease [25]. Hamad [12] studied the clinical phenotypes of 206 children or adults with 16p13.11 duplication and found that 72% exhibited developmental delays, 62% displayed behavioral abnormalities, 27% had certain dysmorphic features, and 8% had congenital heart defects. In this prenatal study, three cases (25.0%, 3/12) revealed cardiovascular system abnormalities, and two cases (16.7%, 2/12) showed widened lateral ventricles, indicating that these are the main clinical phenotypes of fetuses with 16p13.11 duplication. There is a significant difference between the clinical phenotypes observed prenatally and those observed postnatally. Additionally, we discovered a previously unreported case of 16p13.11 duplication that included clinical manifestations such as widened lateral ventricles, tricuspid regurgitation, and right ear malformation. This case further underscores the complexity and diversity of fetal 16p13.11 duplication phenotypes.

The uncertain prognosis of fetuses with 16p13.11 deletions or duplications is one of the main factors influencing decisions to terminate pregnancies [20]. Since 16p13.11 deletions and duplications are associated with incomplete penetrance and a wide range of phenotypes, predicting the postnatal phenotype of the fetus is challenging [3]. Consequently, despite the low expressivity, some couples may opt to terminate the pregnancy to avoid potential risks. Research reports indicate that mothers carrying the 16p13.11 copy number variation are more likely to pass it on to their offspring than fathers [6]. Our study found that among the 14 cases of 16p13.11 deletion, five were inherited from the mother and one from the father, aligning with previous research findings. However, of the 12 cases of 16p13.11 duplication, four were inherited from the mother and four from the father. This difference may be attributed to the fact that previous data primarily came from children and adolescents with developmental delays or intellectual disabilities, whereas our data came entirely from prenatal fetuses. In this study, 18 out of 26 cases chose to continue their pregnancy and gave birth to babies without obvious abnormalities. However, during the follow‐up period, three children with deletions and two children with duplications were found to have neuropsychiatric abnormalities. Given the close association between neurodevelopmental abnormalities and 16p13.11 deletions and duplications, it is possible that more cases of neurodevelopmental abnormalities will be identified as follow‐up studies continue. Parents are often particularly sensitive to abnormalities in facial features, limbs, organs, and other ultrasonic structures, or to severe intellectual disabilities, and may choose to terminate the pregnancy [26]. However, there is generally insufficient attention paid to conditions like 16p13.11 deletion or duplication, which are closely associated with neurodevelopmental abnormalities [27]. Therefore, genetic counseling for these conditions needs to be emphasized. Through pedigree analysis to identify related phenotypes among family members, comprehensive genetic counseling should be provided. This not only helps to prevent excessive termination of pregnancies but also ensures that pregnant women are fully informed about the potential clinical outcomes for the fetus.

The main advantage of this study is that we conducted a comprehensive analysis of the pregnancy information, SNP array results, ultrasound phenotype, and follow‐up information of 26 fetuses with 16p13.11 deletions and duplications. However, there are still some limitations. Firstly, the duration of follow‐up varies. The majority of newborns in this cohort are still very young, making it difficult to effectively assess and diagnose cases of neurodevelopmental abnormalities. Secondly, although the sample size is larger than previous studies focusing on case reports, as a retrospective study, the amount of data is still limited, which may lead to biased conclusions.

## 6 Conclusion

In this study, we expanded the clinical phenotype spectrum of fetuses with 16p13.11 deletions and duplications from a single prenatal center at tertiary hospitals. We conducted a preliminary evaluation of the prenatal ultrasound findings and monitored the postpartum clinical phenotypes during follow‐up. We discovered that the primary manifestations in fetuses with 16p13.11 deletions and duplications may include cardiovascular abnormalities and enlargement of the lateral ventricles. Additionally, the penetrance of these deletions appears to be higher than that of duplications. Furthermore, it is crucial to pay close attention to the neurological development of newborns after birth. Further research is necessary, including accumulating a larger sample size and extending the follow‐up period, to provide more reliable data for clinical doctors and patients.

## Author Contributions

All authors have materially participated in this study and manuscript preparation. Xiaojin Luo, Liping Wu, and Hongyan Niu collected all clinical data and carried out all the molecular and cytogenetic analyses. Ruchun Huang and Fei Zhou participated in the data analysis and drafted the manuscript. Jinshuang Song and Jinmao Xu collected and analyzed ultrasound findings. Yuanyuan Pei, Weiqiang Liu, and Fengxiang Wei designed the work and drafted and revised the manuscript. All authors have approved the final article.

## Ethics Statement

This study was approved (LGFYYXLL‐028) by the Ethics Committee of Longgang District Maternity & Child Healthcare Hospital of Shenzhen City, Shenzhen, Guangdong Province, 518,172, China. All participants gave written informed consent to take part in the present study.

## Consent

The authors have nothing to report.

## Conflicts of Interest

The authors declare no conflicts of interest.

## Data Availability

The data that support the findings of this study are available on request from the corresponding author. The data are not publicly available due to privacy or ethical restrictions.
